# Anti-Acne Activity of Italian Medicinal Plants Used for Skin Infection

**DOI:** 10.3389/fphar.2016.00425

**Published:** 2016-11-10

**Authors:** Kate Nelson, James T. Lyles, Tracy Li, Alessandro Saitta, Eugenia Addie-Noye, Paula Tyler, Cassandra L. Quave

**Affiliations:** ^1^Department of Dermatology, Emory University School of MedicineAtlanta, GA, USA; ^2^Center for the Study of Human Health, Emory University College of Arts and SciencesAtlanta, GA, USA; ^3^Department of Agricultural and Forest Sciences, Università Degli Studi di PalermoPalermo, Italy; ^4^Department of Chemistry, Emory University College of Arts and SciencesAtlanta, GA, USA; ^5^Antibiotic Resistance Center, Emory UniversityAtlanta, GA, USA

**Keywords:** biofilms, antimicrobials, antibiotics, acne, medicinal plants, *Propionibacterium acnes*, ethnopharmacology

## Abstract

*Propionibacterium acnes* is implicated in the pathogenesis of acne vulgaris, which impacts >85% of teenagers. Novel therapies are in high demand and an ethnopharmacological approach to discovering new plant sources of anti-acne therapeutics could contribute to filling this void in effective therapies. The aims of our study were two-fold: (1) To determine if species identified in ethnopharmacological field studies as having traditional uses for skin and soft tissue infection (SSTI) exhibit significantly more activity against *P. acnes* than species with no such reported use; and (2) Chemically characterize active extracts and assess their suitability for future investigation. Extracts of Italian medicinal (for acne and other skin infection) and randomly collected plants and fungi were screened for growth-inhibitory and anti-biofilm activity in *P. acnes* using broth microdilution methods. Bioactive extracts were chemically characterized by HPLC and examined for cytotoxicity against human keratinocytes (HaCaTs). Following evaluation of 157 extracts from 10 fungi and 58 plants, we identified crude extracts from seven species exhibiting growth inhibitory activity (MICs 64–256 μg mL^−1^). All active extracts were examined for cytotoxicity against HaCaTs; extracts from one fungal and one plant species were toxic (IC_50_ 256 μg mL^−1^). HPLC analysis with chemical standards revealed many of these extracts contained chlorogenic acid, p-coumaric acid, ellagic acid, gallic acid, and tannic acid. In conclusion, species used in traditional medicine for the skin exhibited significantly greater (*p* < 0.05) growth inhibitory and biofilm eradication activity than random species, supporting the validity of an ethnobotanical approach to identifying new therapeutics. The anti-acne activity of three extracts is reported for the first time: *Vitis vinifera* leaves, *Asphodelus microcarpus* leaves, and *Vicia sativa* aerial parts.

## Introduction

Acne vulgaris is a common skin disorder affecting more than 85% of teenagers in the United States (James et al., 2009). While it is not a lethal or debilitating disorder, it can be painful and disfiguring, causing significant physiological distress, and heavy economic burden. Acne is associated with increases in anxiety, depression, and suicidal ideation (Dunn et al., 2011). In 2001, a report estimated that over $1 billion is spent each year in the US on acne related health care visits and acne therapies (Lehmann et al., 2001). However, these therapies are not cures, but rather ways of managing this follicular disorder.

Current treatments fall into either two categories: topical or oral. Common topical treatments include benzoyl peroxide, retinoids, and antibiotics (i.e., erythromycin or clindamycin), and common oral treatments include retinoids and antibiotics (i.e., tetracycline and macrolides; Layton, 2016; Zaenglein et al., 2016). In cases of severe acne, combinational treatments are used, usually employing benzoyl peroxide, retinoids, and/or antibiotics together (Layton, 2016; Zaenglein et al., 2016). Antibiotics have been used for over 50 years to treat acne and today one course of topical or systemic treatment typically lasts 3–6 months (Walsh et al., 2016). Antibiotics are thought to inhibit inflammation indicative of acne when used topically and systemically, as well as target *P. acnes* when used topically (Walsh et al., 2016). Like many other bacteria, *P. acnes* is also subject to emerging antibiotic resistance and novel therapies are in high demand worldwide (Sardana et al., 2015; Del Rosso and Zeichner, 2016; Walsh et al., 2016). Pathogenesis targets, such as quorum sensing and biofilms, are organism specific and could limit resistance development (LaSarre and Federle, 2013).

Biofilms contribute to the recalcitrant nature of acne by presenting a physical barrier to which few molecules are capable of penetrating, allowing the bacteria within to persist and cause infection (Donlan, 2001; Vlassova et al., 2011). Biofilms have been implicated in implant-related and chronic infections (Donlan, 2001; Aubin et al., 2014), accounting for an estimated 80% of microbial infections in the body (Sun et al., 2013). *P. acnes* has been detected in biofilms associated with bone, joint and spinal prosthetics, breast implants, external ventricular shunts, and, in rare instances, cardiac devices (Aubin et al., 2014). *P. acnes* biofilms are associated with pathogenesis of acne, with extensive biofilm growth being associated with acne vulgaris (Jahns and Alexeyev, 2014). The high incidence of antibiotic resistance seen in *P. acnes* worldwide is thought to be due to biofilms, as well as the high use of antibiotics (Sardana et al., 2015; Walsh et al., 2016). Only a few studies have evaluated potential *P. acnes* anti-biofilm compounds (Brackman et al., 2014; Sivasankar et al., 2016) and to the best of our knowledge only one study has evaluated medicinal plants for anti-biofilm activity (Coenye et al., 2012). Medicinal plants, in particular, may present a unique source of new therapeutic options. Many studies have been dedicated to documentation of traditional uses of medicinal plants for managing dermatological conditions, and these may represent a strong starting point for drug discovery research on this topic (Pieroni et al., 2004b; Quave et al., 2008b; Cavero et al., 2013; Mabona and Van Vuuren, 2013; Afolayan et al., 2014; Lall and Kishore, 2014).

An ethnobotanical approach for drug discovery uses traditional medicine, historical texts or traditional healers, as a guide toward potentially medicinal plant species. A number of studies addressing different biological targets have demonstrated that an ethnobotanical approach to identifying bioactive compounds in plants is more efficacious than a random search. More than 15 years ago, Slish et al. (1999) compared plant species of which 31 had an ethnobotanical use and 32 were random collections. The random collection extracts had no vasorelaxation activity, but 19% of ethnobotanical extracts did (Slish et al., 1999). Another study focused on the search for antifungal activity compared 114 extracts with antifungal ethnobotanical use and 183 extracts with either no ethnobotanical indication or non-fungal ethnobotanical use. In a series of bioassays, extracts in the ethnobotanical indication group consistently had higher hit rates with the antifungal indicated extracts; 40% had antifungal activity against at least one fungus vs. 21% of plants with no anti-fungal ethnobotanical use (Svetaz et al., 2010). Similarly, a study comparing three groups, skin and soft tissue infection (SSTI) botanicals, non-skin botanicals, and random plants, for their *Staphylococcus aureus* antibacterial and anti-biofilm actions found no difference between the three groups for antibacterial activity, but SSTI ethnobotanicals had more anti-biofilm potential in *S. aureus* models than the random collections (Quave et al., 2008a). Investigating antibacterial bioactivities beyond bacteriostatic or bactericidal action, such as pathogenesis factors, could reveal other mechanisms through which plant secondary metabolites impact pathogen survival and fitness, increasing the chance of discovering new therapeutics. For the purposes of clarity, here we use SSTI to refer to infections of the skin and soft tissues ranging from mild to severe, including those that are purulent (acne, furuncles, abscesses, carbuncles) to non-purulent (necrotizing infection, cellulitis, erysipelas) in nature.

Our research group has already demonstrated interesting anti-staphylococcal activity (in terms of growth, biofilm, and quorum sensing inhibitory action) in the Quave Natural Products Library (QNPL), which includes a collection of plant and fungal extracts derived from natural ingredients used specifically in the treatment of SSTIs—caused by a number of skin pathogenic bacteria, including *Propionibacterium acnes*—and other health concerns in the Mediterranean and other parts of the world (Quave et al., 2012, 2015). Extracts from Italian species in the QNPL are uniquely tied to ethnopharmacological data documented in field studies conducted by Quave and colleagues, and the detailed reports of ethnopharmacological uses of these species have been previously published (Pieroni et al., 2002, 2004a,b; Pieroni and Quave, 2005; Quave et al., 2008b).

The QNPL has only been tested against a limited number of pathogens responsible for SSTIs, and has never been screened for activity against *P. acnes*. In this study, we compare the activity of Italian plants and fungi in the QNPL that were identified as being used in traditional medicine for the treatment of acne and other skin infections to a random collection of Italian species with no reported topical applications for skin infection or inflammation. The overall aim of this study is to compare activity of these two groups through examination of their anti-acne potential against planktonic and biofilm-associated *P. acnes* and evaluate active extracts for future investigation. We hypothesize that extracts from medicinal plants or fungi used for the traditional treatment of SSTIs will demonstrate greater anti-bacterial activity against *P. acnes* (in terms of killing planktonic cells and eradicating biofilms) than those with no reported application to the skin.

## Materials and methods

### Plant and fungal materials

Plant materials were collected in Spring-Summer (May–August) over a period of 2006–2013 from the Vulture Alto-Bradano region of Basilicata, Italy and imported to the United States (US) under US Department of Agriculture permits (PCIP-14-00388; PDEP-09-0228; DP63438). All specimens were collected following the World Health Organization (WHO) guidance on the collection of wild medicinal plants (WHO, 2003). All collections were made on private lands with permission of landowners. Voucher specimens were deposited at the Herbarium Lucanum (HLUC) at the Universitá della Basilicata in Potenza, Italy, and the Emory University Herbarium (GEO) in Atlanta, GA, USA. Identification of species followed the standard Italian flora (Pignatti, 2002), and family assignments followed the Angiosperm Phylogeny Group III guidance (Tropicos.Org, 2015). Bulk materials were separated by part (e.g., leaves, stems, flowers, roots), cut into small pieces with clippers, and then dried in a plant drier with a heat source (35°C) and fan for 48–72 h, until completely dried. Samples were packed with silica packets and vacuum sealed before shipping to the lab for extraction and analysis. Upon arrival, plant materials were ground into a fine powder using a Wiley mill (2 mm mesh size; Wiley Scientific).

Fungi were collected in December 2013 and April–November 2014 from locations in Sicily and imported to the US under USDA permit (P526P-14-02182). Fungal collections were made in broad-leaved and coniferous forests, preserved in paper bags and then transported to the laboratory, dried, and identified. Identification of fungi was carried out using fresh or dried sporomes using European monographs (Ryvarden and Gilbertson, 1993–1994). Species nomenclature followed the Index Fungorum (IF, 2015). Bulk materials were dried and processed as described above. Retention vouchers of the fungal material are stored in the Quave lab.

Extracts from species included in this study were sorted into groups based on history of traditional use for or not for acne and other SSTI treatments, as documented in previously published findings from ethnopharmacological field studies conducted in Italy (Pieroni et al., 2002, 2004a,b; Pieroni and Quave, 2005; Quave et al., 2008b). Importantly, these prior studies included reports of voucher specimens, use-citations, and detailed data on local preparation and application of the natural remedies.

### Natural product extractions

Crude plant extracts were made as macerations in either 95% EtOH or 100% MeOH with a ratio of 1 g (plant material):10 mL organic solvent for two successive periods of 72 h, with daily agitation. Filtered extracts were combined and concentrated at reduced pressure with rotary evaporators (<40°C water bath temperature), shell frozen, and then lyophilized. Extracts were then suspended in DMSO at a stock concentration of 10 mg mL^−1^ prior to bioassay testing, with DMSO representing <1.28% of the final well volume for all tests. Aqueous extracts were created by boiling ground plant material in dH_2_O for 20 min (ratio of 1 g:10 mL), followed by filtration, rotary evaporation and lyophilization as described above. Aqueous extracts were then prepared at a stock concentration of 10 mg mL^−1^ in dH_2_O and filtered at 0.2 μm prior to use in bioassays. Fungal extracts were prepared in 100% MeOH or dH_2_O by double sonication at room temperature for 20 min at a ratio of 1 g:10 mL, followed by filtration, concentration and stock preparation as described for plant extracts. In addition to the crude extracts, we also included bioactive fractions 220D-F2 (from *Rubus ulmifolius*) and 224C-F2 (from *Castanea sativa*) in the panel of tested extracts. The methods for creation of these refined fractions have been previously published (Quave et al., 2012, 2015).

### Microbial strains and culture conditions

The *P. acnes* isolate was obtained from the American Type Culture Collection (ATCC® 6919™). The strain was streaked from freezer stock onto Tryptic soy agar plates (TSA) supplemented with 5% sheep blood (BD, Franklin Lakes, NJ) and incubated at 37°C for 72 h prior to making liquid cultures in brain heart infusion (BHI) broth (BD, Franklin Lakes, NJ) supplemented with 1% dextrose (Fisher Scientific, Waltham, MA) (additional 72 h incubation) for use in the below described assays. All cultures were grown in anaerobic chambers using the EZ Anaerobe Chamber (BD, Franklin Lakes, NJ) and GasPak EZ Anaerobe container sachets (BD, Franklin Lakes, NJ).

### Minimum inhibitory concentration (MIC) for growth

There are no established Clinical and Laboratory Standard Institute (CLSI) guidelines for MIC testing in *P. acnes*. Thus, we followed previously described methods with some modifications (Tsai et al., 2010). Briefly, liquid culture was standardized to 5 × 10^7^ CFU mL^−1^ by optical density (0.05 at OD_590nm_) in fresh BHI supplemented with 1% dextrose, and this was confirmed with plate counts. Our initial screen of the QNPL was undertaken in 96-well plates (Greiner Bio-One International, Monroe, NC) at a final well-concentration of 256 μg mL^−1^ (<1.28% DMSO in well) in a total well volume of 200 μL. The OD_600nm_ of plates was read with a Cytation 3 multimode plate reader (BioTek, Winooski, VT) prior to incubating under anaerobic conditions for 72 h at 37°C. A second OD read was taken at 72 h post-inoculation, and the final percent inhibition of growth was determined using a previously reported formula (Quave et al., 2008a) which takes into account any interference due to color of the test extracts. The most active extracts (% inhibition ≥50%) were retested by serial dilution from 2 to 256 μg mL^−1^ to determine concentrations required for MIC_50_ and MIC_90_. The MIC_50_ and MIC_90_ values were defined as the minimum concentration necessary to achieve ≥50 and ≥90% inhibition of growth, respectively.

### Minimum biofilm eradication concentration (MBEC)

A previously described method was followed with modifications (Coenye et al., 2007). Briefly, liquid culture was standardized as described above for MIC testing. Biofilms were established in tissue culture treated U-bottom 96-well plates (TPP, Trasadingen, Switzerland). Following 24 h incubation under anaerobic conditions, media was gently aspirated by manual pipetting and extract treated media or control -resveratrol (MP Biomedicals, Santa Ana, CA), was added. Initial screens were conducted at a final well-concentration of 256 μg mL^−1^ in a total well volume of 100 μL. Established biofilms were exposed to extract treatment for 24 h under anaerobic conditions, and then media was aspirated, wells gently rinsed with PBS, heat-fixed at 37°C for 60 min in an oven and stained with crystal violet (Hardy Diagnostics, Santa Maria, CA) for 15 min. Excess crystal violet was washed off the plates in water and the plates were dried prior to eluting the stain with 10% Tween 80 (2.5% aqueous preparation) in EtOH. The eluate was diluted at a ratio of 1:10 in PBS and the final OD_595nm_ measured with a plate reader. The most active extracts (% inhibition ≥50%) were retested by serial dilution from 2 to 256 μg mL^−1^ to determine MBEC_50_ and MBEC_90_. The MBEC_50_ and MBEC_90_ values were defined as the minimum concentration necessary to achieve ≥50 and ≥90% eradication of attached biofilm, respectively.

### Cytotoxicity analysis

Human immortalized keratinocytes (HaCaT) were used to evaluate the potential skin toxicity of the most active extracts identified via MIC and MBEC testing. Cells were maintained in Dulbecco's modified Eagle's medium with glucose at 4.5 g L^−1^ and L-glutamine (Corning, Corning, NY). Media was supplemented with 10% heat-inactivated fetal bovine serum (Seradigm, Randor, PA) and a solution of 100 IU Penicillin and 100 μg mL^−1^ Streptomycin (Corning, Corning, NY). Cells were cultured in 75 cm^2^ flasks incubated at 37°C with 5% CO_2_. A solution of 0.25% trypsin and 0.1% EDTA in HBSS (Corning, Corning, NY) was used to detach cells from the bottom of the flask before splitting and plating.

After reaching at least 80% confluence in the flask, cells were seeded to a 96-well tissue culture plate (Fisher Scientific, Waltham, MA). Cells were resuspended, counted with a hemocytometer, and standardized to a concentration of 4 × 10^4^ cells per mL (8 × 10^3^ cells per final 200 μL well volume). Plates were incubated for 48 h at 37°C with 5% CO_2_. Following incubation, the medium was aspirated, replaced with fresh medium, and sterile filtered extracts and the vehicle control (DMSO) were serially diluted from 2 to 256 μg mL^−1^. Plates were incubated for 24 h at 37°C with 5% CO_2_. Cytotoxicity was determined with a LDH Assay Kit (G-Biosciences, St. Louis, MO) following manufacturer's protocol. Briefly, after 24 h, 20 μL of lysis buffer was added in triplicate and the plate was incubated for another 45 min. Plates were centrifuged at 1500 rpm for 4 min. Following centrifugation, 50 μL of supernatant from each well was transferred to a new 96-well plate, mixed with 50 μL of substrate mix and incubated for 20 min while being protected from light. After incubation, 50 μL of stop solution was added to all wells, and absorbance was recorded at 490 nm with a plate reader.

### Characterization of active extracts

Preliminary characterization of the most active extracts, which either exhibited a MIC_50_ or MBEC_50_, was pursued by HPLC. The analysis was performed on an Agilent 1260 Infinity system running OpenLab CDS ChemStation (Agilent Technologies, Santa Clara, CA) with an Agilent ZORBAX Eclipse XDB-C18 (250 mm × 4.6 mm, 5 μm) column (Santa Clara, CA) with compatible guard column at a column temperature of 35°C. Mobile phase reagents were HPLC grade and purchased from Fisher Scientific (Waltham, MA), except for the Type 1 water, which was obtained from an EMD Millipore MILLI-Q water system (Billerica, MA). Mobile phase consisted of a linear gradient elution 0.1% formic acid in water (A) and 0.1% formic acid in acetonitrile (B) at a flow rate of 2 mL min^−1^. Initial conditions were 95:5 (A:B) changing to 63:37 (A:B) at 40 min, to 100% B at 80 min and held until 90 min. Samples were prepared in MeOH and 10 μL or 20 μL injections were made. Chromatograms were monitored at 254 nm and 280 nm. Authentic standards were used to aid in peak identification by comparison of retention times and UV profiles. Caffeic acid, chlorogenic acid, p-coumaric acid, ellagic acid, ferulic acid, and resveratrol were purchased from MP Biomedicals (Solon, OH) at ≥98% purity. Gallic acid was purchased from Acros Organics (NJ) at 98% purity. ACS grade tannic acid from Sigma Aldrich (St. Louis, MO) was characterized prior to use by HPLC-PDA analysis.

Liquid chromatography-Fourier transform mass spectrometry (LC-FTMS) was performed on the MeOH extract of *Hapalopilus rutilans* using a Shimadzu SIL-ACHT (Kyoto, Japan) and Dionex 3600SD HPLC pump (Sunnyvale, CA) with the above described chromatographic conditions. The data was acquired in MS^1^ mode scanning from *m/z* 150 to 1500 on a Thermo Scientific LTQ-FT Ultra MS (Waltham, MA) in negative ESI mode and processed with Thermo Scientific Xcalibur 2.2 SP1.48 software (San Jose, CA). The capillary temperature and voltage were 275.0°C and −19.00 V, sheath gas of 60, source voltage 5.0 kV, and source current 100.0 μA.

### Statistical analyses

Percent inhibition of growth and biofilm formation (screening concentration of 256 μg mL^−1^) was compared for extracts sorted into two groups: plants used for SSTI and plants those not documented for this use. GraphPad Prism software was used to conduct statistical tests. A Student's *t*-test (unpaired, 2-tailed), was conducted to compare percent inhibitory activity of the two groups, with *p* < 0.05 considered significant. A second analysis of the data was also performed following the removal of multiple outliers by the ROUT method, which was more suitable to this analysis than the Grubbs' method as it accounts for multiple outliers (Barnett and Lewis, 1994; Motulsky and Brown, 2006). The false discovery rate, or “Q,” was set to 1% for this procedure.

## Results

A total of 10 fungal species, distributed across 10 genera and 6 families, and 58 plant species, distributed across 55 genera and 32 families were extracted and evaluated for growth inhibition and biofilm eradication activity against *P. acnes*. Seventeen of these species have been documented in the ethnobiological literature as being used in therapies for SSTIs (Pieroni et al., 2002, 2004a,b; Pieroni and Quave, 2005; Quave et al., 2008b); these are denoted with a “+” in the SSTI column of Table 1.

**Table 1:** **Minimum inhibitory concentrations (MICs) of plant extracts (μg mL^−1^) against *Propionibacterium acnes* (ATCC 6919)**.

**Taxa [voucher code]**	**Common Name**	**SSTI use^‡^**	**Part Extracted^†^**	**Extraction Solvent**	**MIC_50_**	**MIC_90_**
**FUNGI**						
**Amanitaceae**						
*Amanita caesarea* (Scop.) Pers. [AS-19]	Caesar's mushroom	−	fb	MeOH	−	−
**Ganodermataceae**						
*Ganoderma lucidum* (Curtis) P. Karst. [AS-10]	Lingzhi mushroom	−	fb	MeOH	−	−
				H_2_O	−	−
**Gleophyllaceae**						
*Gloeophyllum sepiarium* (Wulfen) P. Karst. [AS-4]	−	−	fb	MeOH	−	−
**Hymenochaetales**						
*Fuscoporia torulosa* (Pers.) T. Wagner & M. Fisch. [AS-1]	−	−	fb	H_2_O	−	−
*Trichaptum biforme* (Fr.) Ryvarden [AS-12]	−	−	fb	MeOH	−	−
				H_2_O	−	−
**Meripilaceae**						
*Meripilus giganteus* (Pers.) P. Karst. [AS-11]	Giant polypore	−	fb	MeOH	−	−
				H_2_O	−	−
**Polyporaceae**						
*Coriolopsis gallica* (Fr.) Ryvarden [AS-13]	−	−	fb	MeOH	−	−
				H_2_O	−	−
*Fomes fomentarius* (L.) Fr. [AS-4]	Tinder fungus	−	fb	MeOH	−	−
				H_2_O	−	−
*Hapalopilus rutilans* (Pers.) Murrill [AS-5]	Tender nesting polypore	−	fb	MeOH	128	256
				H_2_O	−	−
*Trametes versicolor* (L.) Lloyd [AS-8]	Turkey tail	−	fb	MeOH	−	−
				H_2_O	−	−
**PLANTAE**						
**Adoxaceae**						
*Sambucus ebulus* L. [CQ-180]	Dwarf Elder	+	infl	EtOH	−	−
				MeOH	−	−
			le	EtOH	−	−
				MeOH	−	−
			st	EtOH	−	−
*Sambucus nigra* L. [CQ-151]	Elderberry	+	fr	EtOH	−	−
				MeOH	−	−
			le	EtOH	−	−
				MeOH	−	−
			wp	EtOH	−	−
			infl	MeOH	−	−
**Amaryllidaceae**						
*Allium cepa* L. [CQ-206]	Onion	+	le; bu	EtOH	−	−
				MeOH	−	−
**Apiaceae**						
*Daucus carota* L. [CQ-215]	Queen Anne's lace	−	le; st	EtOH	−	−
				MeOH	−	−
			infl; infr	EtOH	−	−
*Ferula communis* L. [CQ-214]	Giant fennel	−	fr	EtOH	−	−
			st	EtOH	−	−
				MeOH	−	−
			infl	MeOH	−	−
			ro	MeOH	−	−
*Foeniculum vulgare* subsp. *piperitum* Cout. [CQ-192]	Fennel	−	le; st	EtOH	−	−
*Foeniculum vulgare* subsp. *vulgare* [CQ-196]	Fennel	−	le; st	EtOH	−	−
				MeOH	−	−
**Apocynaceae**						
*Vinca major* L. [CQ-139]	Bigleaf periwinkle	−	le; st; ro	MeOH	−	−
**Araceae**						
*Arum italicum* Mill. [CQ-175]	Italian lords and ladies	−	st	EtOH	−	−
			fr	EtOH	−	−
			sta	EtOH	−	−
			le	EtOH	−	−
				MeOH	−	−
**Asparagaceae**						
*Leopoldia comosa* (L.) Parl. [CQ-105]	Tassel hyacinth	−	le; infl	MeOH	−	−
			bu	MeOH	−	−
**Asteraceae**						
*Achillea millefolium* L. [CQ-176]	Common yarrow	+	infl	EtOH	−	−
			le; st	EtOH	−	−
			le; st; fl	EtOH	−	−
				MEOH	−	−
*Anacyclus tomentosus* (Gouan) DC. [CQ-167]	Whitebuttons	−	le; st; fl	EtOH	−	−
*Cichorium intybus* L. [CQ-161]	Chicory	−	le; st; fl	EtOH	−	−
*Scolymus hispanicus* L. [CQ-199]	Common goldenthistle	−	le; st; fl	EtOH	−	−
				MeOH	−	−
*Tussilago farfara* L. [CQ-202]	Coltsfoot	−	le; st; ro	EtOH	−	−
			le; st; fl	MeOH	−	−
**Brassicaceae**						
*Cardaria draba* (L.) Desv. [CQ-108]	Whitetop	−	fl; st; le; ro	MeOH	−	−
**Caprifoliaceae**						
*Dipsacus fullonum* L. [CQ-201]	Fuller's teasel	−	le; st	EtOH	−	−
				MeOH	−	−
			fl	EtOH	−	−
*Knautia arvensis* J.M. Coult. [CQ-190]	Field scabiosa	−	le;st;fl	EtOH	−	−
*Knautia lucana* Lacaita & Szabó [CQ-166]	−	−	le; st; fl	EtOH	−	−
				MeOH	−	−
*Lonicera alpigena* L. [CQ-213]	Alpine honeysuckle	−	wp	EtOH	−	−
				MeOH	−	−
			le	EtOH	−	−
**Caryophyllaceae**						
*Saponaria officinalis* L. [CQ-210]	Bouncingbet	−	le; st; fl	EtOH	−	−
				MeOH	−	−
**Dennstaedtiaceae**						
*Pteridium aquilinum* (L.) Kuhn [CQ-211]	Western brackenfern	−	st	MeOH	−	−
**Fabaceae**						
*Acacia dealbata* Link [CQ-115]	Silver wattle	−	infl	MeOH	−	−
			le	MeOH	−	−
			st	MeOH	−	−
*Anthyllis vulneraria* L. [CQ-147]	Common kidneyvetch	−	le; st; fl	EtOH	−	−
				MeOH	−	−
*Astragalus monspessulanus* L. [CQ-112]	Montpellier milk vetch	−	fl; le; ro; st	EtOH	−	−
				MeOH	−	−
*Coronilla emerus* L. [CQ-137]	Scorpion senna	−	wst	EtOH	−	−
				MeOH	−	−
			le; fl	MeOH	−	−
*Melilotus albus* Medik. [CQ-193]	Sweetclover	−	le; st; fl	EtOH	−	−
				MeOH	−	−
*Trifolium* spp. [CQ-185]	−	−	le; st; fl	EtOH	−	−
				MeOH	−	−
*Robinia pseudoacacia* L. [CQ-155]	Black locust	−	le	MeOH	−	−
*Vicia sativa* subsp. *sativa* [CQ-119]	Garden vetch	−	le; st; fl	MeOH	128	256
**Fagaceae**						
*Castanea sativa* Mill. [CQ-191]	Sweet Chestnut	+	le	EtOH	256	−
				MeOH	256	−
				^*^Fraction 224C-F2	256	256
				H_2_O	−	−
			gle	EtOH	−	−
				MeOH	256	−
			wp	EtOH	−	−
				MeOH	128	−
*Quercus cerris* L. [CQ-228; CQ-301]	European turkey oak	−	st; fr	EtOH	−	−
			le	MeOH	−	−
**Gentianaceae**						
*Centaurium pulchellum* (Sw.) Druce [CQ-217]	Branched centaury	−	le; ro; st; fl	EtOH	−	−
**Hypericaceae**						
*Hypericum perforatum* L. [CQ-183]	St. John's Wort	+	le; st; fl	EtOH	−	−
				MeOH	−	−
**Juglandaceae**						
*Juglans regia* L. [CQ-181]	Walnut	+	le	EtOH	−	−
				MeOH	−	−
			wp	EtOH	256	256
				MeOH	128	256
			imfr	MeOH	−	−
**Juncaceae**						
*Juncus articulatus* L. [CQ-216]	Jointleaf rush	−	le; fr	EtOH	−	−
**Lamiaceae**						
*Ballota nigra* L. [CQ-160]	Black horehound	+	ro	MeOH	−	−
			le	MeOH	−	−
			st	MeOH	−	−
*Marrubium vulgare* L. [CQ-170]	Horehound	+	ro	MeOH	−	−
			le; st; fl	MeOH	−	−
*Mentha spicata* L. [CQ-224]	Spearmint	−	le; st; fl	EtOH	−	−
*Rosmarinus officinalis* L. [CQ-113]	Rosemary	+	le; st; fl	MeOH	128	256
**Liliaceae**						
*Lilium candidum* L. [CQ-174]	Madonna lily	−	infl	EtOH	−	−
**Malvaceae**						
*Malva sylvestris* L. [CQ-156]	Common mallow	+	le	MeOH	−	−
			st	MeOH	−	−
**Moraceae**						
*Ficus carica* L. [CQ-173]	Edible fig	+	le	EtOH	−	−
			wp	EtOH	−	−
				MeOH	−	−
			imfr	MeOH	−	−
**Nyctaginaceae**						
*Mirabilis jalapa* L. [CQ-222]	Four o'clock flower	−	le; fl; fr	EtOH	−	−
				MeOH	−	−
**Oleaceae**						
*Olea europaea* L. [CQ-197]	Olive	−	wp	EtOH	−	−
				MeOH	−	−
			le	MeOH	−	−
**Plantaginaceae**						
*Plantago major* L. [CQ-225]	Boradleaf plantain	+	le; ro; fl; st	EtOH	−	−
				MeOH	−	−
**Poaceae**						
*Agropyron repens* (L.) P. Beauv. [CQ-208]	Couch grass	−	le; st; ro	EtOH	−	−
*Arundo donax* L. [CQ-146]	Giant reed	+	le; st	MeOH	−	−
			in	MeOH	−	−
**Ranunculaceae**						
*Delphinium fissum* Waldst. [CQ-187]	−	−	le; st; fl; fr	EtOH	−	−
*Ranunculus acris* L. [CQ-135]	Meadow buttercup	−	le; st; fl	MeOH	−	−
**Rosaceae**						
*Prunus spinosa* L. [CQ-163]	Blackthorn	−	wp; le	EtOH	−	−
			fr	MeOH	−	−
*Rosa canina* var. *canina* [CQ-152]	Dog rose		wp	MeOH	−	−
				80% MeOH	−	−
			ga	MeOH	−	−
			le	80% MeOH	−	−
			fr	MeOH	−	−
			le; fl	80% MeOH	−	−
*Rubus ulmifolius* Schott.	Elmleaf blackberry	+	ro	EtOH	−	−
				^*^Fraction 220D-F2	−	−
				MeOH	−	−
			wst	EtOH	−	−
**Rubiaceae**						
*Galium verum* L. [CQ-177]	Yellow bedstraw	+	le; st; fl	EtOH	−	−
				MeOH	−	−
**Scrophulariaceae**						
*Verbascum thapsus* L. [CQ-172]	Great mullein	−	le	MeOH	−	−
**Ulmaceae**						
*Ulmus minor* Mill. [CQ-195]	Field elm	−	wp	EtOH	−	−
				MeOH	−	−
			le	MeOH	−	−
**Vitaceae**						
*Vitis vinifera* var. *aglianico* [CQ-209]	Grape vine	−	le	MeOH	64	64
**Xanthorrhoeaceae**						
*Asphodelus microcarpus* Salzm. & Viv. [CQ-109]	−	+	infl	MeOH	−	−
			le	MeOH	128	128
**ANTIBIOTIC CONTROLS**						
Clindamycin					0.25	2
Erythromycin					0.063	2
Resveratrol					100	100

Part Extracted^†^: bu, bulb; fl, flowers; gle, gall infected leaves; imfr, immature fruit; in, inter nodes; infl, inflorescence; infr, infructescence; fb, fruiting body; ga, galls; le, leaves; ro, roots; st, stems; sta, stalk; wp, woody parts; wst, woody stem. SSTI use^‡^: “+” indicates that there is a documented ethnobotanical use for skin and soft tissue infections (SSTI) or skin inflammations for this species; “−“ indicates no documented use for SSTIs. ^*^The extraction protocol for 224C-F2 and 220D-F2 have been previously published (Quave et al., 2012, 2015). The MIC for growth is represented at MIC_50_ (or at 50% inhibition of the growth control) or MIC_90_ for 90% inhibition. “−“ indicates that the MIC_50_ was not detected in the concentration range tested (2-256 μg mL^−1^). None of the extracts tested exhibited MBEC_50_ within the tested concentration range, and thus this column is omitted from the table.

### Anti-bacterial activity

#### Growth inhibition

MIC_50_ and MIC_90_ values were detected for one fungus (Hapalopilus rutilans) and 6 plants (*Vicia sativa* subsp. sativa, *C. sativa, Juglans regia, Rosmarinus officinalis, Vitis vinifera* var. *aglianico*, and *Asphodelus microcarpus*) at the tested range (2–256 μg mL^−1^). The extraction method and plant or fungal tissue(s) extracted for each species, as well as all MICs for extracts and controls are reported in Table 1.

The most active extract in terms of growth inhibition included a leaf extract of *V. vinifera* var. *aglianico*, with a MIC_50_ and MIC_90_ value of 64 μg mL^−1^. Extracts with MIC_50_ values of 128 μg mL^−1^ included *Hapalopilus rutilans* (MeOH extract of fruiting body), *V. sativa* subsp. *sativa* (MeOH extract of aerial parts), *J. regia* (MeOH extract of woody parts), *R. officinalis* (MeOH extract of aerial parts) and *A. microcarpus* (MeOH extract of leaves). Several extracts derived from the leaves and woody parts of *C. sativa* also exhibited MIC_50_ values ranging from 128 to 256 μg mL^−1^. Positive controls clindamycin and erythromycin exhibited MIC_50_ of 0.25 μg mL^−1^ and 0.063 μg mL^−1^, respectively, and MIC_90_ of 2 μg mL^−1^, while resveratrol exhibited an MIC_50_ and MIC_90_ of 100 μg mL^−1^.

#### Biofilm eradication

None of the extracts tested exhibited at least 50% eradication of mature biofilm within the tested concentration range. The positive control for biofilm eradication (resveratrol) exhibited MBEC_50_ and MBEC_90_ of 100 μg mL^−1^.

#### Comparison of activity based on traditional use

**Figure 1:**
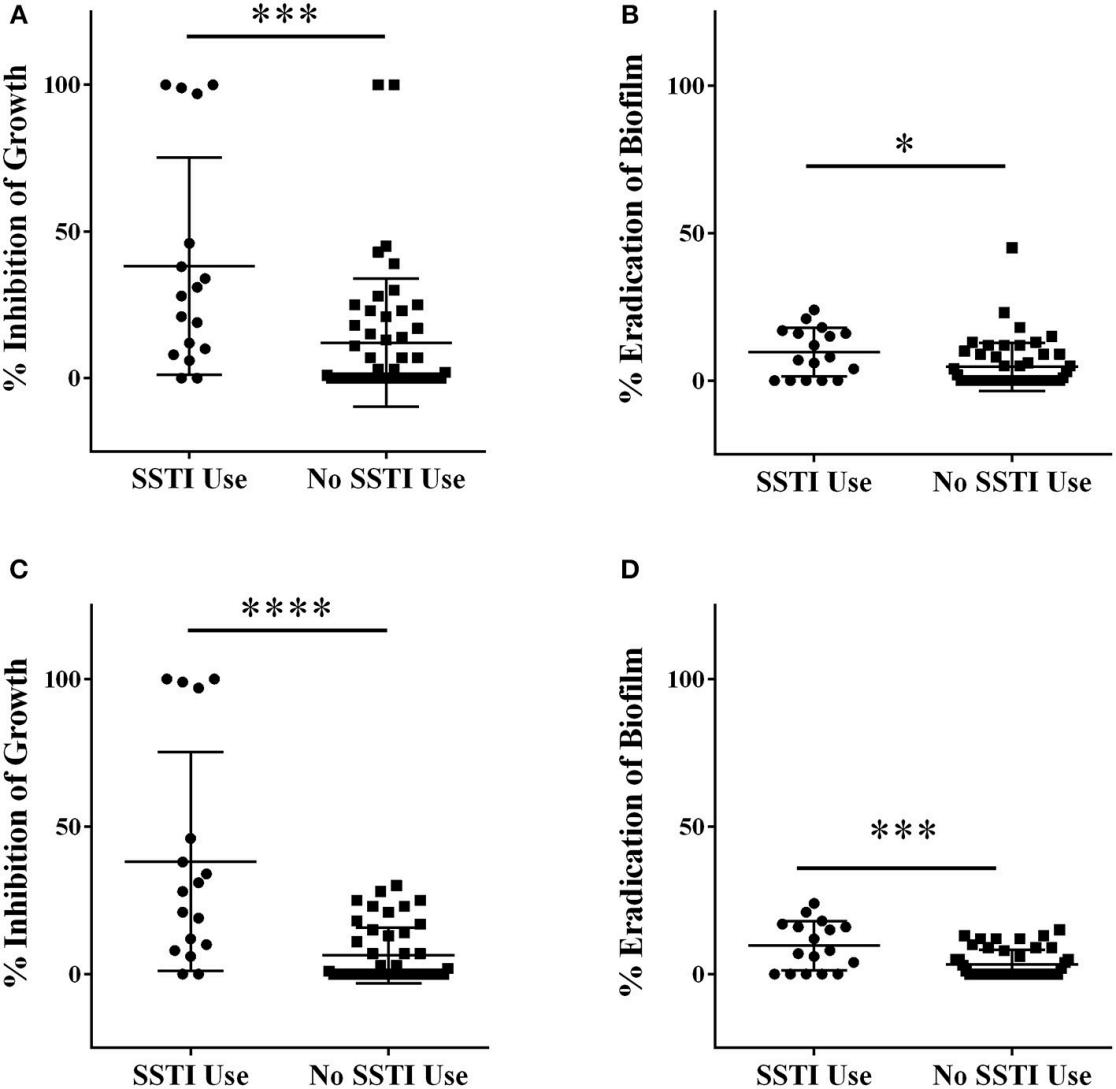
**Extracts were screened for percent inhibitory activity against (A)** growth and **(B)** biofilm at a concentration of 256 μg mL^−1^. Outlier data points were identified using the ROUT method, removed and the data-set reanalyzed for **(C)** growth and **(D)** biofilm activity. ^*^p < 0.05; ^**^p < 0.01; ^***^p < 0.001; ^****^p < 0.0001.

Initial screening of Italian plants and fungi in the QNPL was conducted at 256 μg mL^−1^, resulting in percent inhibition values for both growth and biofilm. Extracts were sorted into two groups, based on history of traditional medicinal use for acne and other SSTIs in Italy. Extracts from plants used in traditional therapies for skin infections exhibited a mean value of 38% inhibition of growth, which was significantly greater (*p* < 0.001) than that of extracts from randomly selected plants and fungi (12%), Figure 1A. Likewise, there was a significant difference (*p* < 0.05) in biofilm eradicating activity between both groups (10% in SSTI versus 5% in random, Figure 1B). Outliers in the data set were also identified and removed by the ROUT method and then reassessed using a Student's *t*-test. Five outliers were identified and removed from the growth inhibitory data, and the groups were found to be significantly different (*p* < 0.0001), Figure 1C. Three outliers were identified and removed in the biofilm datasets, also reconfirming a significant difference between groups (*p* < 0.001), Figure 1D.

### Mammalian cytotoxicity

**Figure 2:**
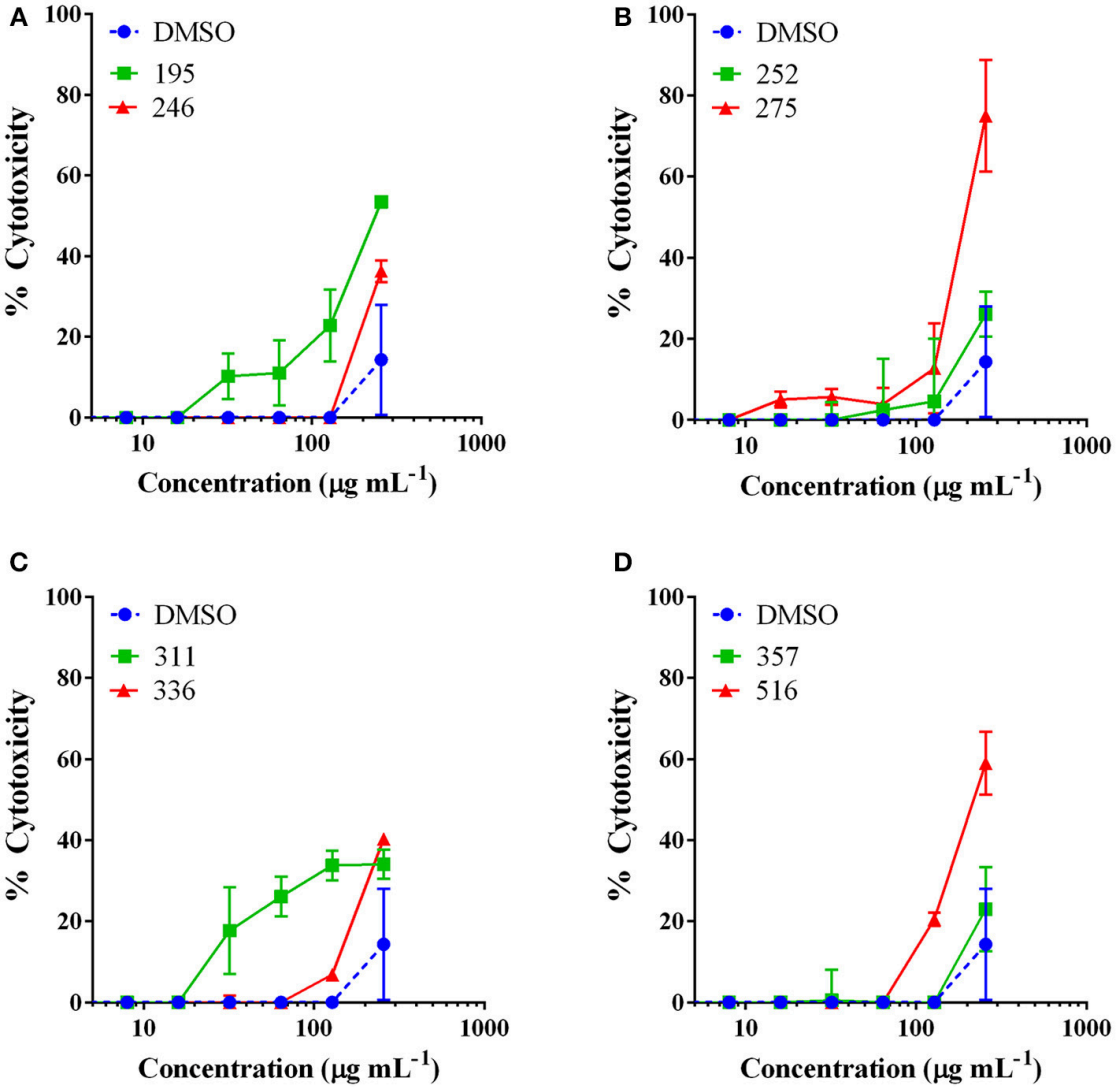
**The most active extracts were examined for mammalian cytotoxicity against an immortalized line of human keratinocytes (HaCaT)**. Data are reported as percent cytotoxicity and the vehicle (DMSO) was included as a control. Extract numbers correspond as follows: **(A) 195**: *Juglans regia* (EtOH extract of woody parts); **246**: *Asphodelus microcarpus* (MeOH extract of leaves). **(B) 252**: *Castanea sativa* (MeOH extract of woody parts); and **275**: *Juglans regia* (MeOH extract of woody parts). **(C)** Extract numbers: **311**: *Rosmarinus officinalis* (MeOH extract of aerial parts: leaves, stems, flowers); **336**: *Vitis vinifera* var. *aglianico* (MeOH extract of leaves). **(D) 357**: *Vicia sativa* subsp. *sativa* (MeOH extract of aerial parts: leaves, stems, flowers); and **516**: *Hapalopilus rutilans* (MeOH extract of fruiting bodies). *Castanea sativa* leaf extracts were found to be non-toxic at the tested range and this data is reported in a previous work (Quave et al., 2015).

Extracts with MIC_50_ values detected were examined for cytotoxicity against an immortalized line of human keratinocytes at a test range of 2–256 μg mL^−1^ in order to determine whether the observed antibacterial activity was due to specific antibacterial action or general toxicity. The only extracts to exhibit an IC_50_ came from *J. regia* and *Hapalopilus rutilans*, both at the highest test dose of 256 μg mL^−1^ (Figure 2). All other extracts tested exhibited limited to no toxicity at the concentrations necessary for antibacterial activity.

### Chemical characterization of active extracts

We used a panel of ubiquitous chemical standards in effort to provide basic chemical characterization of extracts of interest. The most bioactive extracts were examined for the presence of caffeic acid (**1**), chlorogenic acid (**2**), p-coumaric acid (**3**), ellagic acid (**4**), ferulic acid (**5**), gallic acid (**6**), resveratrol (**7**), and tannic acid (**8**) by comparison with authentic standards by HPLC-DAD. The presence or absence of these standards in extracts is reported in Table 2. Several extracts derived from *C. sativa* were active as growth inhibitors for *P. acnes*. Ellagic acid (**4**) and gallic acid **(6)** were both detected in all of the *C. sativa* extracts (Supplementary Figure 1B). More detailed chemical characterization of the most active extract fraction, 224C-F2, is reported in a recent publication concerning its anti-virulence effects against *S. aureus* (Quave et al., 2015).

**Table 2:** **Chemical characterization of the most bioactive extracts**.

**Source**	**Extract**	**Extraction detail**	**1**	**2**	**3**	**4**	**5**	**6**	**7**	**8**
*Asphodelus microcarpus* Salzm. & Viv.	246	MeOH extract of leaves	−	+	+	+	−	−	−	−
*Castanea sativa* Mill.	134	EtOH extract of leaves	−	−	−	+	−	+	−	−
	224	MeOH extract of leaves	−	−	−	+	−	+	−	−
	224C	Ethyl acetate partition of 224	−	−	−	+	−	+	−	−
	224C−F2	Flash chromatography fraction of 224C	−	−	−	+	−	+	−	−
	252	MeOH extract of woody parts	−	−	−	+	−	+	−	−
*Hapalopilus rutilans* (Pers.) Murrill	516	MeOH extract of fruiting bodies	−	−	−	−	−	−	−	−
*Juglans regia* L.	195	EtOH extract of woody parts	−	−	−	+	−	−	−	+
	275	MeOH extract of woody parts	−	−	−	−	−	−	−	−
*Rosmarinus officinalis* L.	311	MeOH extract of aerial parts (leaves, stems, flowers)	−	−	−	−	−	−	−	−
*Vicia sativa* subsp. *sativa*	357	MeOH extract of aerial parts (leaves, stems, flowers)	−	−	−	−	−	+	−	+
*Vitis vinifera* var. *aglianico*	336	MeOH extract of leaves	−	−	+	+	−	+	−	+

*Samples were examined by HPLC and compared to the following standards: caffeic acid (**1**), chlorogenic acid (**2**), p-coumaric acid (**3**), ellagic acid (**4**), ferulic acid (**5**), gallic acid (**6**), resveratrol (**7**), tannic acid (**8**). Compounds identified in the extract are denoted by “+”, those absent by “−“. Chromatograms are reported in Supplementary Figure 1 and structures in Supplementary Figure 2*.

The *H. rutilans* extract underwent further characterization using negative ESI LC-FTMS. The [M-H]^−^ for **9** was determined to be 291.06755, providing an empirical formula of C_18_H_11_O_4_(Δ 2.365). Comparison with published literature identified **9** as the antitumor and dihydroorotate dehydrogenase inhibitor polyporic acid, which can account for up to 40% of the fungal fruiting body's mass (Kraft et al., 1998).

## Discussion

The central aim of this study was to determine whether or not extracts that are derived from plants or fungi used in topical skin therapeutics for acne and other forms of SSTI in traditional medicine would have greater activity against *P. acnes* than specimens from a random sample. Our hypothesis was supported; we found that extracts of plants used for traditional anti-infective skin therapeutics had a significantly greater impact on planktonic growth and on mature biofilms of *P. acnes* than extracts from random samples (including medicinal plants used for other purposes unrelated to the skin). Here, we discuss some of the specific sources of bioactivity reported in this study and make recommendations for future investigation.

Of the 157 extracts screened in this study, 12 exhibited more than 50% growth inhibition at the test concentration range. These twelve came from one fungus and six plant species. The bioactive fungal species is a polypore fungus, and is also known by the common names of tender nesting polypore, purple dye polypore, or the cinnamon bracket. While some other polypore fungi (i.e., *Trametes versicolor*, also known as Turkey tail) can play an important role as cosmetic or cosmeceutical ingredients (Hyde et al., 2010), to the best of our knowledge, there are no reports of such uses for *H. rutilans*. Although nothing has been reported concerning topical toxicity or contact dermatitis with this species, multiple reports on poisoning events following consumption of this fungus have been reported, and include symptoms such as purple discoloration of the urine, nausea, vomiting, blurred visions, hallucinations, and even death (Kraft et al., 1998; Villa et al., 2013). Such neurotoxic effects have been attributed to the high content of polyporic acid in the fungus, which was also identified as the principal component of the extract evaluated in the present study (65.74% of the crude extract, representing 5.7% of dry mass of the fruiting body). While the extract of this species inhibited *P. acnes* growth (MIC_50_ 128 μg mL^−1^) and inhibited mature biofilm by 45% at a concentration of 256 μg mL^−1^, it also exhibited cytotoxicity to skin cells (HaCaT) (IC_50_ of 256 μg mL^−1^) at or near concentrations inhibitory to *P. acnes*, suggesting that the activity observed is due to general toxicity of the extract.

While none of the plant species examined were effective at eradicating mature *P. acnes* biofilm, we did identify five species with growth inhibitory activity at concentrations that were non-toxic to HaCaTs. The most active was a leaf extract from the *aglianico* grape varietal (*V. vinifera* var. *aglianico*) with a MIC_50_ and MIC_90_ of 64 μg mL^−1^. No cytotoxicity was detected against HaCaT cells at that concentration and only 40% cytotoxicity was reached at the highest concentration tested (256 μg mL^−1^). Among the standards included in our chemical characterization studies, **3**, **4**, **6**, and **8** were all detected in this extract. It is unlikely that **4**, **6**, or **8** were individually responsible for the growth inhibitory activity observed as the MIC for these against another strain of *P. acnes* was reported as 800, 1800, and 1000 μg mL^−1^, respectively (Patil et al., 2012). While **7** was not detected in this leaf extract, it is known to be found in grapevine, particularly the grape skin. It has been identified as having growth inhibitory and biofilm-eradicating activity against *P. acnes* (Coenye et al., 2012). This is the first report of grape leaf extracts exhibiting growth inhibitory effects. The interactions between grapevine and *P. acnes* are quite interesting, especially considering recent phylogenetic and population genetic research that suggests a horizontal interkingdom transfer of this human opportunistic pathogen to grapevine as an obligate endophyte during the period in which this species was domesticated by humans (Campisano et al., 2014). Further studies on anti-acne activity of this extract and its individual constituents are warranted.

Another active extract came from *V. sativa* subsp. *sativa* (Garden vetch), and it exhibited a MIC_50_ and MIC_90_ against *P. acnes* at concentrations of 128 and 256 μg mL^−1^, respectively. The IC_50_ for cytotoxicity to skin cells (HaCaT) was not detectable at this test range. HPLC analysis revealed low levels of **6** and **8**, as well as a number of larger peaks that should be prioritized for identification in future studies. Garden vetch is most widely used as fodder for livestock (esp. for horses and cattle). It has been previously examined for antibacterial, anti-biofilm, and quorum-sensing inhibitory effects against *S. aureus*, with no detected activity (Quave et al., 2008a, 2011). Further investigation of this non-toxic and abundantly available fodder crop as a potential acne therapy is merited.

### Bioactivity of medicinal plants used in traditional treatment of SSTI

The remaining four active species of interest—*C. sativa* (Sweet Chestnut), *J. regia* (Walnut), *R. officinalis* (Rosemary), and *A. microcarpus*—exhibited MIC_50_ values of 128–256 μg mL^−1^ and all have indications for traditional use in the topical treatment of skin infections and inflammations as documented in ethnobotanical studies conducted in Italy (e.g., Pieroni et al., 2002, 2004a,b; Quave et al., 2008b).

Different traditional preparations of Sweet Chestnut (*C. sativa*) have been documented in ethnopharmacological studies in Italy for topical applications as an emollient, anti-inflammatory, skin-whitener, and anti-eczema treatment. The antibacterial activity of chestnut extracts has been reported against a number of pathogens (Basile et al., 2000), and more recently, attention has shifted to activity in blocking virulence pathways in *S. aureus* (Quave et al., 2015). The non-toxic nature of the chestnut leaf extracts assessed here, along with its combined role in inhibiting both staphylococcal virulence and growth of *P. acnes* makes it an interesting candidate for further analysis.

Walnut trees (*J. regia*), on the other hand, have been documented for topical use as an anti-fungal (particularly for fungal infections of the feet), antiseptic, compress, and as a colorant (for hair). The cytotoxicity (IC_50_ of 256 μg mL^−1^) of this particular walnut extract against skin cells (HaCaT) suggests general toxicity, and thus is not a high priority for further study.

Rosemary *(R. officinalis*) is used traditionally as a hair wash, facial toner, foot bath and general skin tonic. Previous research on this specific rosemary extract demonstrated both mild growth inhibitory (MIC_50_ of 512 μg mL^−1^) and biofilm inhibitory (MBIC_50_ of 64 μg mL^−1^) activity against *S. aureus* (Quave et al., 2008a). Inhibition of delta-toxin production (IC_50_ of 64 μg mL^−1^), a virulence factor controlled by *S. aureus* quorum sensing, was also detected for this extract (Quave et al., 2011). The growth inhibitory activity of rosemary extracts and essential oils against *P. acnes* has been reported in a number of studies (Fu et al., 2007; Weckesser et al., 2007; Tsai et al., 2013). Due to its common incorporation as an ingredient in skin and hair care ingredients, further investigation of its anti-acne activity is warranted.

Lastly, *A. microcarpus* also demonstrated growth inhibitory activity without notable toxicity to keratinocytes. It has been reported in the ethnobotanical literature for use as a diuretic, for otitis and toothache in Algeria (Sarri et al., 2014), a wild food source in the Medieval Levant (Rubin, 2002) and a skin emollient, lenitive and treatment for lung diseases in Sardinia (Loi et al., 2005). Extracts and individual compounds extracted from the tubers of *A. microcarpus* have demonstrated antimicrobial (against *S. aureus* and *Cryptococcus neoformans*) and anti-malarial (against *Plasmodium falciparum*) activities (Ghoneim et al., 2013), but this is the first study to document its antibacterial properties against *P. acnes*. Due to its favorable therapeutic index (activity:toxicity), this species also merits further investigation for potential anti-acne applications.

## Conclusions

In conclusion, we have demonstrated that plants and fungi used in the traditional treatment of acne and other SSTIs exhibit higher levels of activity than plants with no relevant ethno-use, thus validating the ethnobotanical approach as a useful drug discovery tool. Specifically, we found a statistically significant difference in growth inhibition and biofilm eradication of *P. acnes* by plants and fungi used in traditional medicine to treat SSTIs in comparison to a random sample of plants and fungi with other unrelated (or no) human uses. Furthermore, we identified seven species with growth inhibitory activity. However, two of these were determined to be cytotoxic. An additional two have already been reported in the literature for their anti-acne activity (Fu et al., 2007; Weckesser et al., 2007; Coenye et al., 2012; Tsai et al., 2013). This study was the first to document the growth inhibitory activity of organic extracts of *V. sativa* aerial parts, *V. vinifera* leaves and *A. microcarpus* leaves. Further research on the bioactivity of these extracts and their individual constituents are planned in future work as there is a need for new therapeutics for *P. acnes*.

## Author contributions

KN and CQ wrote the manuscript. AS collected and assigned taxonomic identities to fungal samples. CQ collected and identified all plant samples. PT performed chemical extractions of fungal samples. TL, EA-N, and KN performed plant extractions and microbiological and cell culture experiments. JL performed chemical and chromatographic analyses on the active extracts. CQ, KN, and JL performed data analyses. All authors read, revised, and approved of the final manuscript.

## Funding

This work was supported by the Bionorica SE Global Research Initiative (Project title: Botanical natural product inhibitors of acne biofilms). The funding agency had no role in the design, implementation or analysis of the study.

### Conflict of interest statement

The authors declare that the research was conducted in the absence of any commercial or financial relationships that could be construed as a potential conflict of interest.

## Supplementary material

Supplementary Figure 1 **UV-Vis chromatograms of bioactive extracts with standards identified**. Standards shown here include chlorogenic acid (**2**), p-coumaric acid (**3**), ellagic acid (**4**), gallic acid (**6**) and tannic acid (**8**). Polyporic acid (**9**) was identified by LC-FTMS. **(A)** Extract numbers correspond as follows. **195**: *Juglans regia* (EtOH extract of woody parts); **246**: *Asphodelus microcarpus* (MeOH extract of leaves); **252**: *Castanea sativa* (MeOH extract of woody parts); and **275**: *Juglans regia* (MeOH extract of woody parts); **311**: *Rosmarinus officinalis* (MeOH extract of aerial parts: leaves, stems, flowers); **336**: *Vitis vinifera* var. *aglianico* (MeOH extract of leaves); **357**: *Vicia sativa* subsp. *sativa* (MeOH extract of aerial parts: leaves, stems, flowers); and **516**: *Hapalopilus rutilans* (MeOH extract of fruiting bodies). **(B)** A number of different extracts derived from a single species, *Castanea sativa*, were evaluated. Extract numbers correspond as follows. **134**: EtOH extract of leaves; **226**: MeOH extract of gall-infected leaves; **224**: MeOH extract of leaves; **224C**: ethyl acetate partition of 224; **224C-F2**: flash chromatography fraction of 224C; **252**: MeOH of woody parts.

Supplementary Figure 2 **Compounds identified in the bioactive extracts, as reported in Table 2 and Supplementary Figure 1: chlorogenic acid (2), p-coumaric acid (3), ellagic acid (4), gallic acid (6), tannic acid (8), and polyporic acid (9)**.
