# Supplementary figures and images for: Anti-Acne Activity of Italian Medicinal Plants Used for Skin Infection
Source: Front Pharmacol. 2016 Nov 10;7:425. doi: 10.3389/fphar.2016.00425 (PMC5103262; doi:10.3389/fphar.2016.00425)

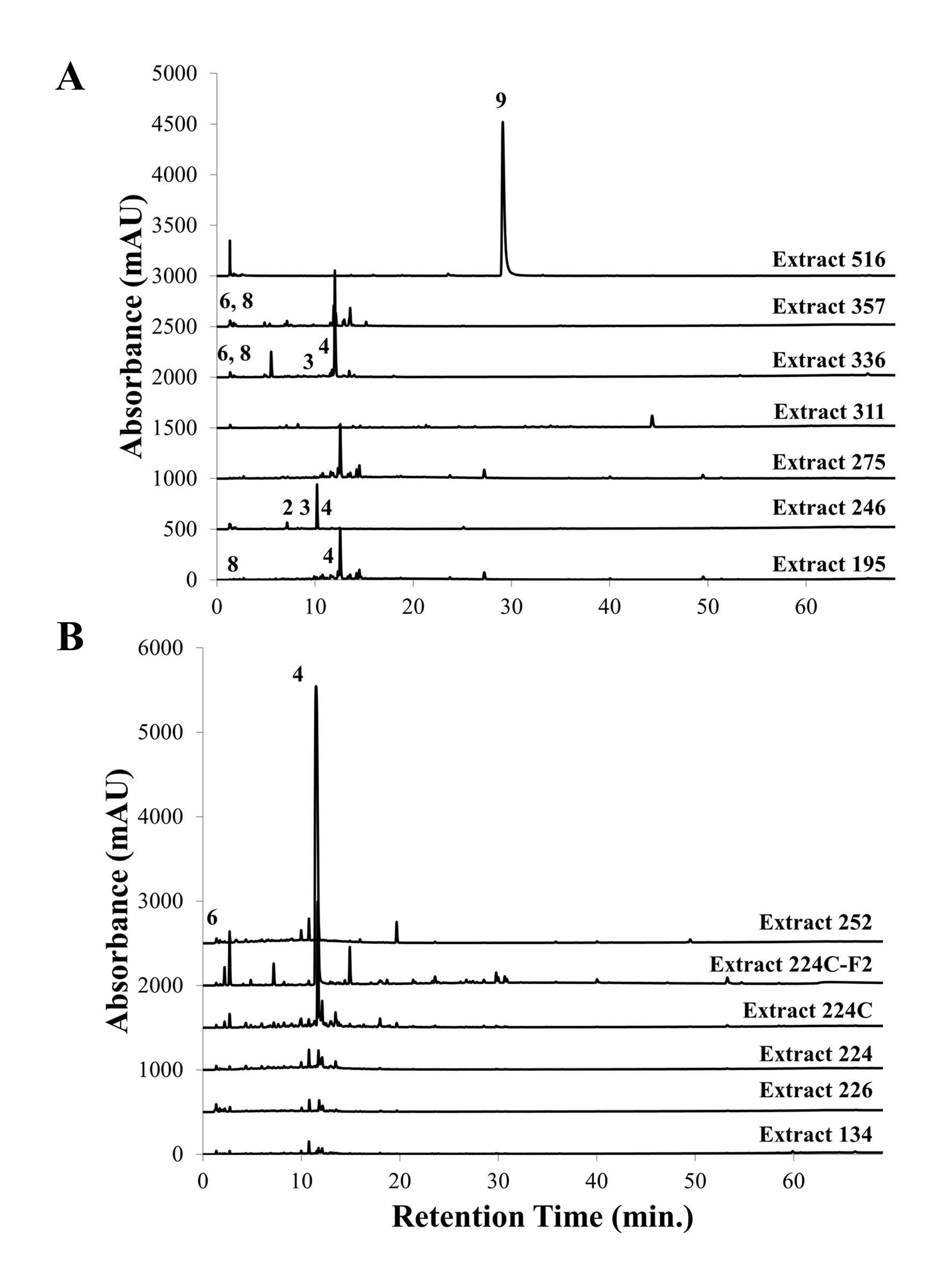

Supplement: Supplementary Figure 1 — UV-Vis chromatograms of bioactive extracts with standards identified. Standards shown here include chlorogenic acid (2), p-coumaric acid (3), ellagic acid (4), gallic acid (6) and tannic acid (8). Polyporic acid (9) was identified by LC-FTMS. (A) Extract numbers correspond as follows. 195: Juglans regia (EtOH extract of woody parts); 246: Asphodelus microcarpus (MeOH extract of leaves); 252: Castanea sativa (MeOH extract of woody parts); and 275: Juglans regia (MeOH extract of woody parts); 311: Rosmarinus officinalis (MeOH extract of aerial parts: leaves, stems, flowers); 336: Vitis vinifera var. aglianico (MeOH extract of leaves); 357: Vicia sativa subsp. sativa (MeOH extract of aerial parts: leaves, stems, flowers); and 516: Hapalopilus rutilans (MeOH extract of fruiting bodies). (B) A number of different extracts derived from a single species, Castanea sativa, were evaluated. Extract numbers correspond as follows. 134: EtOH extract of leaves; 226: MeOH extract of gall-infected leaves; 224: MeOH extract of leaves; 224C: ethyl acetate partition of 224; 224C-F2: flash chromatography fraction of 224C; 252: MeOH of woody parts. [file Image1.TIF]

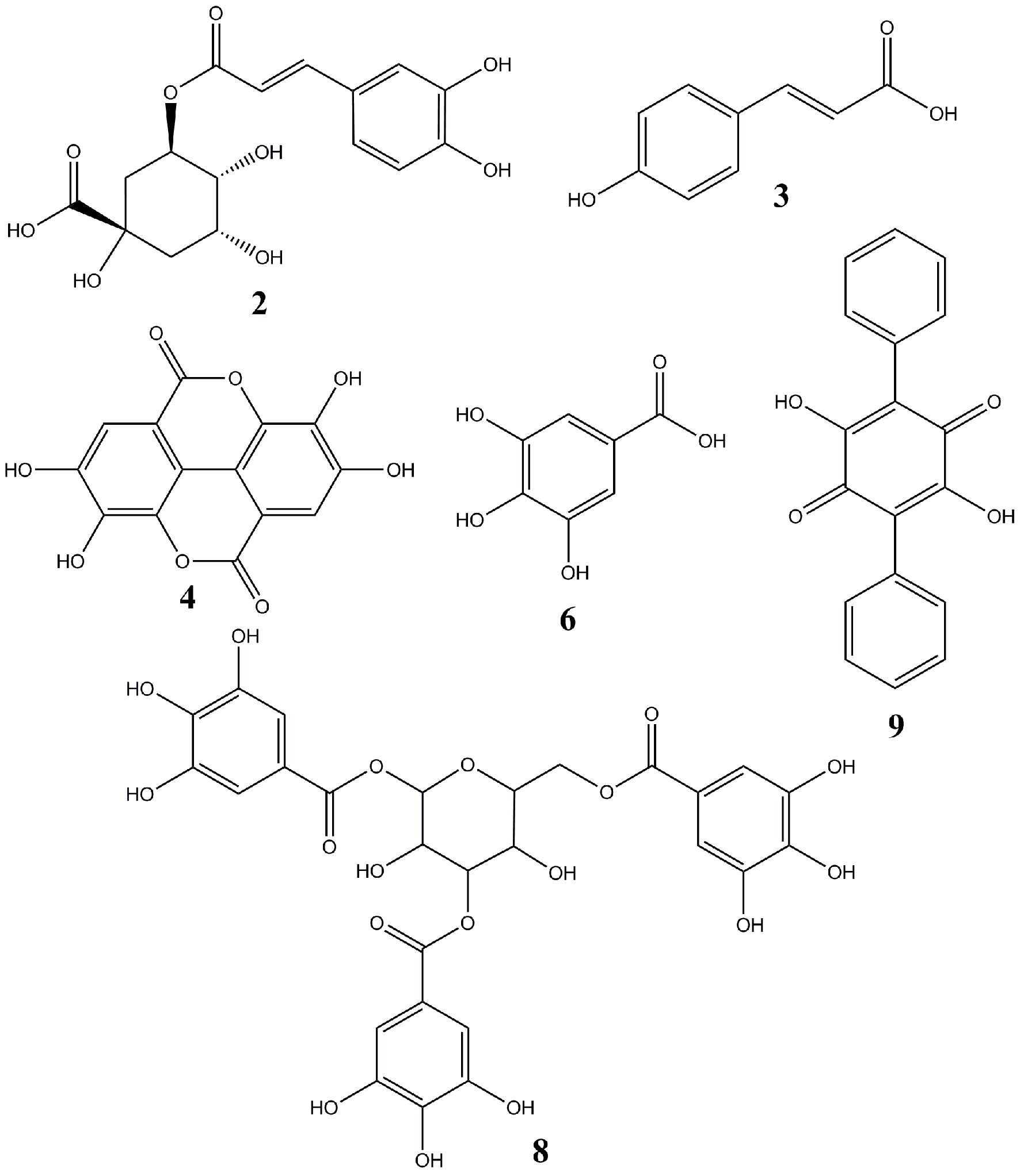

Supplement: Supplementary Figure 2 — Compounds identified in the bioactive extracts, as reported in Table 2 and Supplementary Figure 1: chlorogenic acid (2), p-coumaric acid (3), ellagic acid (4), gallic acid (6), tannic acid (8), and polyporic acid (9). [file Image2.TIF]
